# Current Status of Canine Melanoma Diagnosis and Therapy: Report From a Colloquium on Canine Melanoma Organized by ABROVET (Brazilian Association of Veterinary Oncology)

**DOI:** 10.3389/fvets.2021.707025

**Published:** 2021-08-16

**Authors:** Carlos Eduardo Fonseca-Alves, Ênio Ferreira, Cristina de Oliveira Massoco, Bryan Eric Strauss, Wagner José Fávaro, Nelson Durán, Natália Oyafuso da Cruz, Simone Carvalho dos Santos Cunha, Jorge Luiz Costa Castro, Marcelo Monte Mor Rangel, Carlos Henrique Maciel Brunner, Matias Tellado, Denner Santos dos Anjos, Simone Crestoni Fernandes, Andrigo Barbosa de Nardi, Luiz Roberto Biondi, Maria Lucia Zaidan Dagli

**Affiliations:** ^1^Paulista University – UNIP, Bauru, Brazil; ^2^São Paulo State University – UNESP, Botucatu, Brazil; ^3^Department of General Pathology, Institute of Biological Sciences, Federal University of Minas Gerais, Belo Horizonte, Brazil; ^4^Laboratory of Pharmacology and Toxicology, Department of Pathology, School of Veterinary Medicine and Animal Science, University of São Paulo, São Paulo, Brazil; ^5^Laboratório de Vetores Virais, Centro de Investigação Translacional em Oncologia/LIM24, Instituto do Câncer do Estado de São Paulo, Faculdade de Medicina, Universidade de São Paulo, São Paulo, Brazil; ^6^Departamento de Biologia Estrutural e Funcional, Instituto de Biologia, Universidade Estadual de Campinas (UNICAMP), Campinas, Brazil; ^7^Laboratory of Urogenital Carcinogenesis and Immunotherapy, University of Campinas, Campinas, Brazil; ^8^Pet Care Oncologic Center – Radiotherapy, São Paulo, Brazil; ^9^OncoPet Veterinary Hospital, Rio de Janeiro, Brazil; ^10^Pontifical Catholic University of Paraná (PUCPR), Curitiba, Brazil; ^11^Vet Cancer – Animal Oncology and Pathology, São Paulo, Brazil; ^12^Paulsita University (UNIP), São Paulo, Brazil; ^13^Vet Oncologia Cancer Clinic, Buenos Aires, Argentina; ^14^Department of Veterinary Clinic and Surgery, São Paulo State University (UNESP), Jaboticabal, Brazil; ^15^Specialized Service in Veterinary Oncology (SEOVET), São Paulo, Brazil; ^16^Department of Veterinary Clinic and Surgery, Faculty of Agricultural and Veterinary Sciences, São Paulo State University, São Paulo, Brazil; ^17^Metropolitan University of Santos (UNIMES), Santos, Brazil; ^18^Laboratory of Experimental and Comparative Oncology, Department of Pathology, School of Veterinary Medicine and Animal Science, University of São Paulo, São Paulo, Brazil

**Keywords:** oral melanoma, cutaneous melanoma, dog, melanocytic disorders, electrochemotherapy, radiation therapy, immunotherapy

## Introduction

Melanoma is a prevalent, aggressive form of cancer in dogs. New treatment or preventive modalities are necessary to control this disease in dogs. On December 03, 2020, the Brazilian Association of Veterinary Oncology, ABROVET, organized the “Colloquium on Canine Melanoma” to present the newest achievements for the treatment of this disease.

Invited talks included fundamental aspects of canine melanoma, and conventional and innovative therapies. The talks were delivered online and more than 100 attendees joined the transmission. This report aims to present the most important information about canine melanoma discussed at the Colloquium.

### Canine Melanoma: Fundamental Aspects

#### Melanocytic Neoplasms in Dogs: A Pandora's Box, by Carlos Eduardo Fonseca Alves

Canine oral melanoma is one of the most diffuse tumors in dogs worldwide and it is related to a high metastatic rate and poor prognosis. The first publication regarding melanocytic tumors was in 1949 (1), and the first publication of a canine melanoma from oral mucosa was in 1950 (2). After the publication of the first oral melanoma case report (2), several other studies have been performed in order to establish diagnostic, prognostic, and predictive markers.

**Figure 1:**
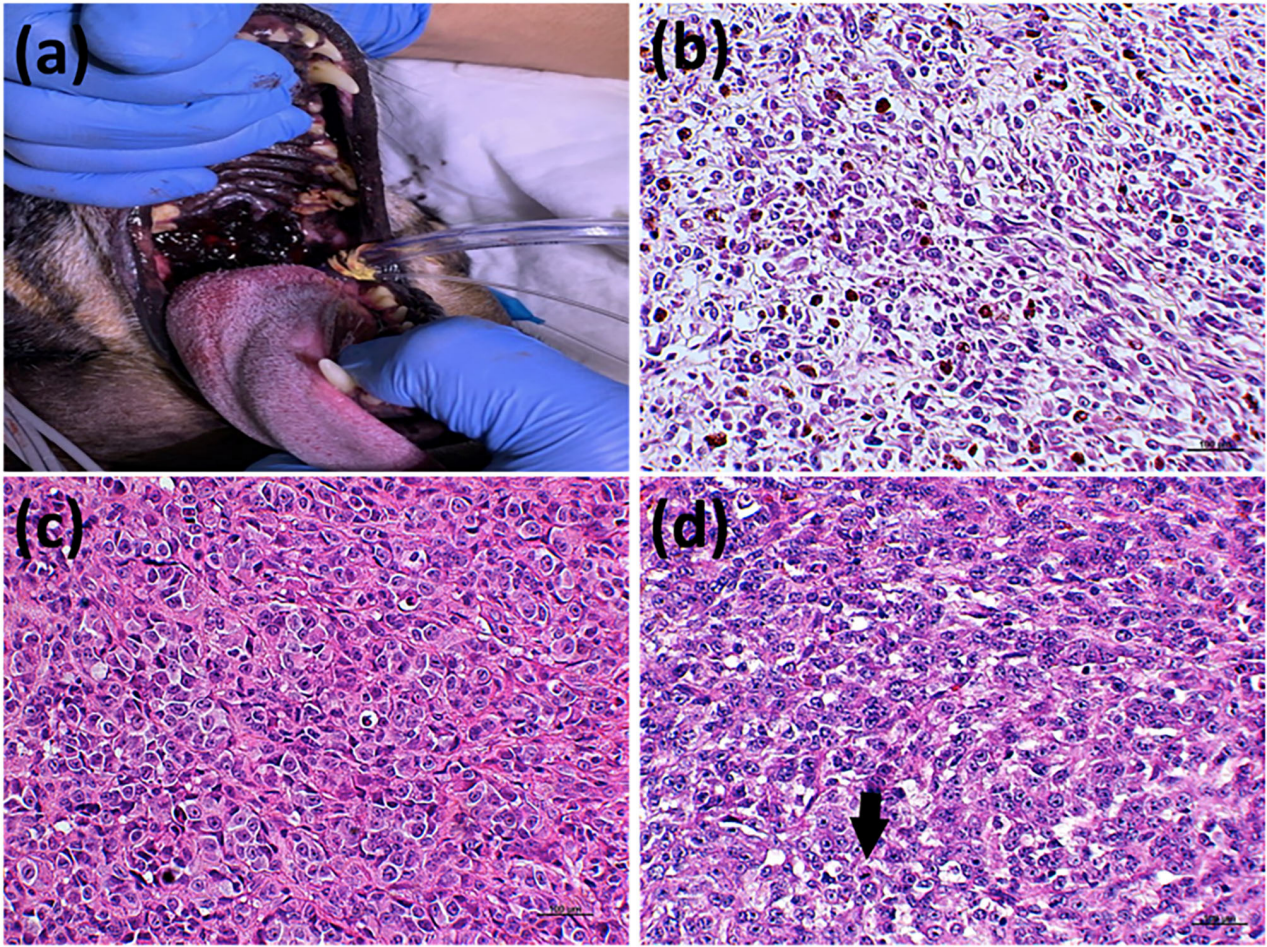
Prognostic criteria for canine oral melanomas. **(a)** Patient with an oral melanoma in the palate with high degree of pigmentation. The degree of pigmentation has a direct 661 relation with patient's prognosis. Patients with heavily pigmented tumors show better outcomes. **(b)** Histological evaluation of a canine oral melanoma with <50% of pigmented cells. **(c)** Histological section of a canine oral melanoma with a marked nuclear atypia, also considered a poor prognostic factor. **(d)** Canine oral melanoma histological section evidencing a mitosis (arrows), one of the most important prognostic factors. Hematoxylin and eosin counterstaining, 40x.

The first important approach for treating dogs with oral melanoma is to establish the prognosis (3). Although several studies have tried to standardize new prognostic molecular factors, the histological features remain pivotal. Recently, the Oncology Pathology Working Group (OPWG), a Veterinary Cancer Society and American College of Veterinary Pathologists initiative, proposed a consensus on the diagnosis of and histopathologic prognostication for canine melanocytic neoplasms (3). Among the histological prognostication criteria proposed in the consensus, nuclear atypia, vascular invasion, and mitotic index were considered in addition to other histological criteria (Figure 1). Interestingly, the degree of pigmentation presented a direct relation with overall survival. Thus, dogs with amelanotic melanoma had a shorter lifespan (4). However, the outcome is not predictable in melanomas with moderate, low, or no pigmentation (3).

**Figure 2:**
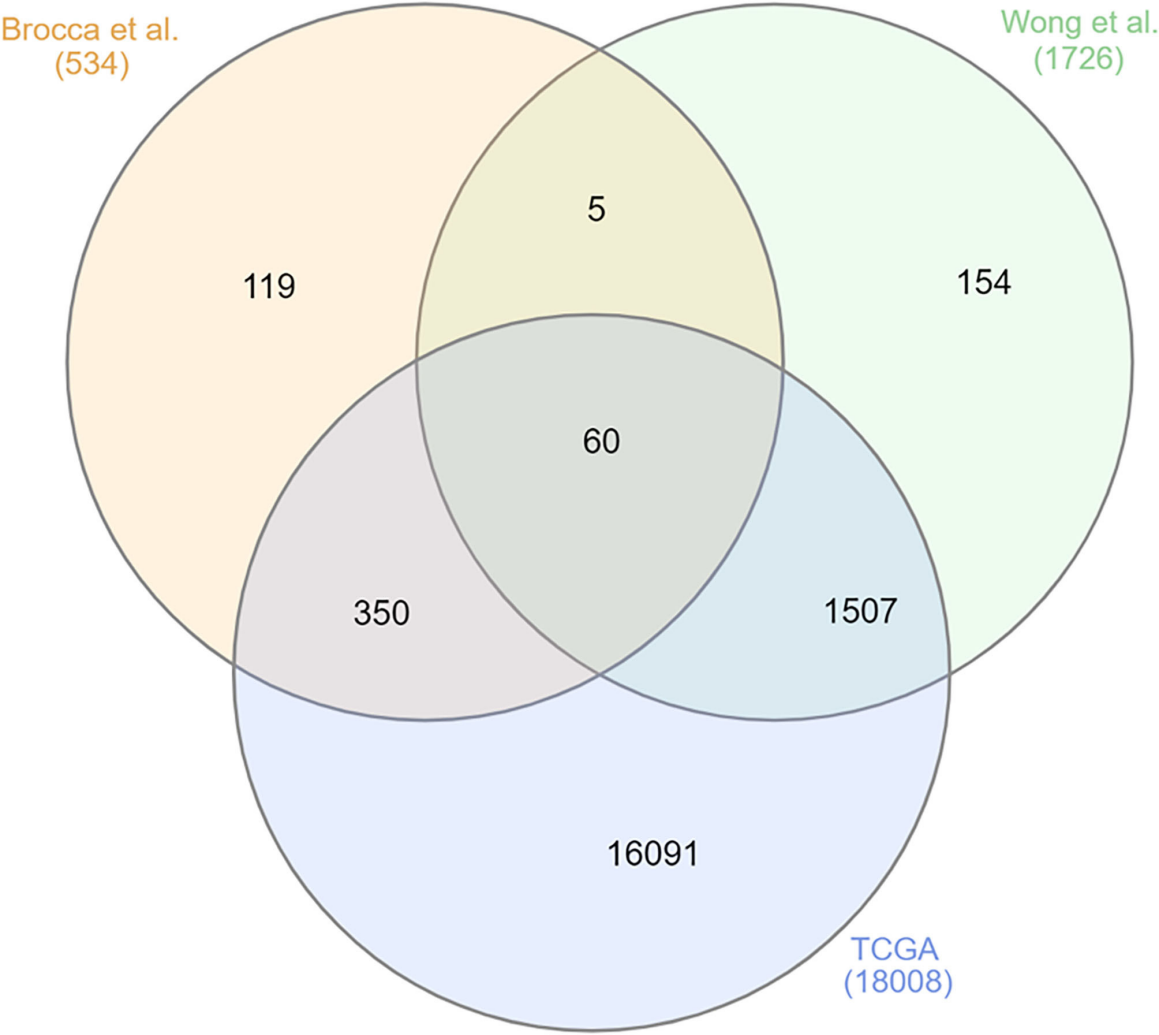
Venn diagram demonstrating the most commonly mutated genes among the three datasets, two canine oral melanoma (8, 9) and one human melanoma dataset (The Cancer Genome Atlas—TCGA 10). Here, 60 commonly mutated genes were identified. Figure generated online (http://www.interactivenn.net/).

Owing to the increasing use of big data in cancer research, recent studies have focused on different molecular prognostic and predictive markers for canine oral melanoma. Among them, somatic focal amplifications on chromosome 30 have been associated with poor patient outcomes. Chromosomal imbalances and transcriptome dysregulations seem to be frequent in canine oral melanoma (5–8). To summarize genomic data associated with melanoma mutations, an integrative *in silico* analysis was performed with the two previous canine oral melanoma studies (9) and the human data available for human melanoma in “The Cancer Genome Atlas” (10). This analysis identified 60 commonly mutated genes among human and canine melanomas (Figure 2).

**Figure 3:**
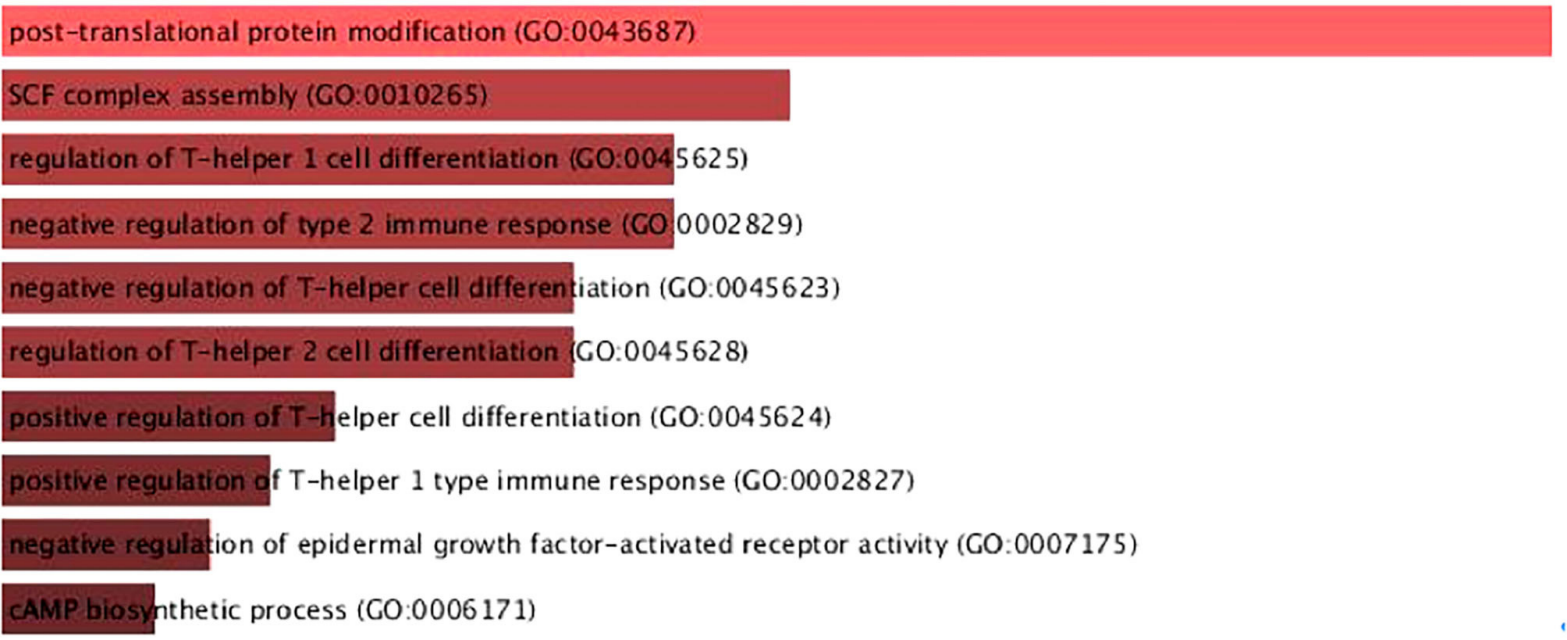
Biological process terms of the 60 dysregulates genes in canine and human melanoma. It is possible to observe differences in processes related to immune system response. Figure generated using the online tool Enrichr [https://maayanlab.cloud/Enrichr/enrich#; (11, 12)].

Most of the 60 dysregulated genes were related to immune response and tyrosine kinase terms, such as negative regulation of T-helper cell differentiation, SCF complex assembly, and activation of protein kinase A activity. For a better visualization, the biological process terms related with the 60 genes are shown in Figure 3 (11, 12).

**Figure 4:**
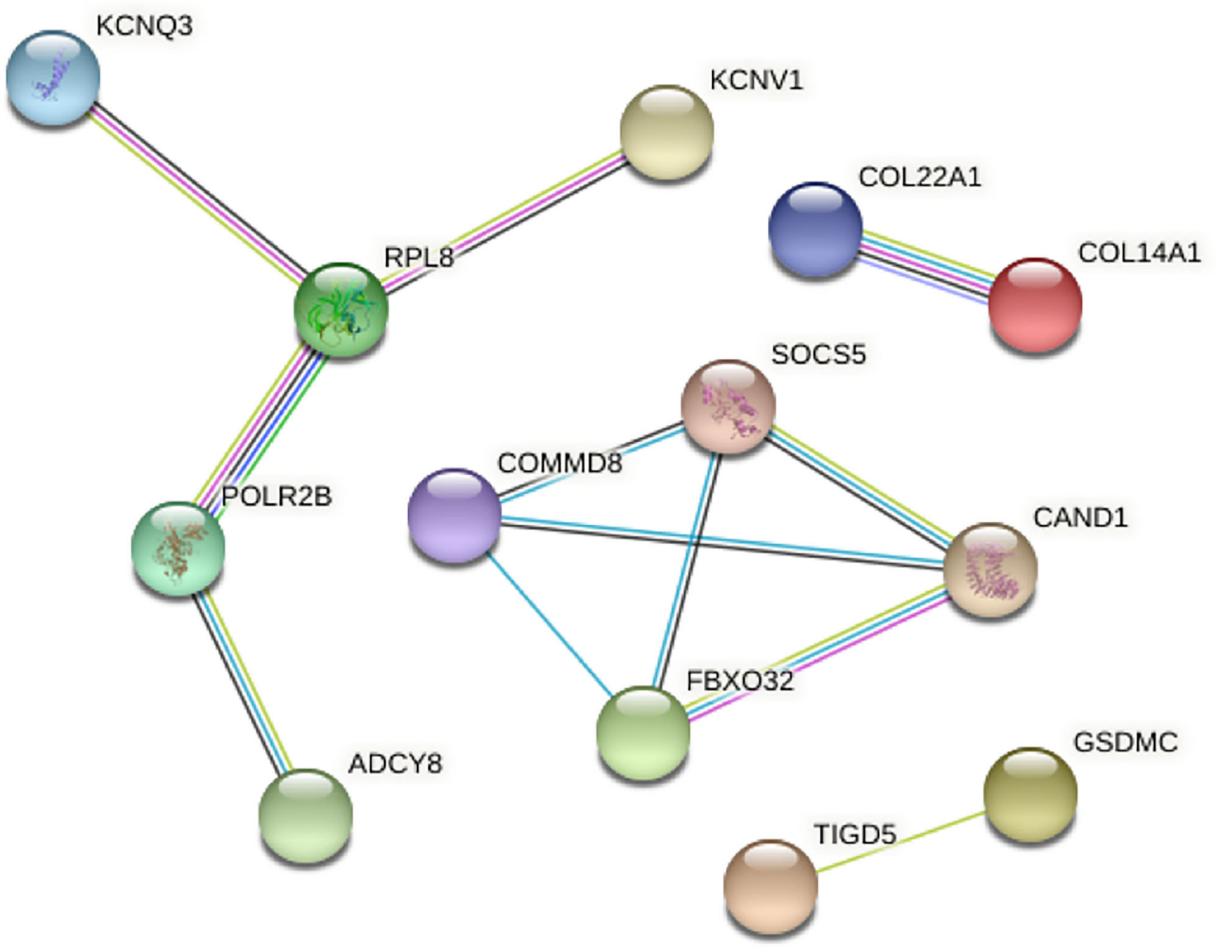
Protein-to-protein interaction analysis with the commonly mutated genes between canine and human melanoma. The disconnected nodes were excluded and four different networks were identified. Figure generated using the online tool STRING (https://string-db.org/).

Among the 60 dysregulated genes in canine and human melanoma, protein-to-protein interaction (PPI) analysis was performed for better visualization of the protein interaction network. In this analysis, the disconnected nodes were excluded, which revealed four independent networks (Figure 4). The genes *RPL8, POLR2B, FBXO32*, and *SOCS5* showed the highest interactions. *RPL8* gene is a ribosomal protein responsible for protein synthesis and *POLR2B* is responsible for encoding the second largest subunit of RNA polymerase II. The *FBXO32* gene is related to mediation of ubiquitination and degradation of target proteins and *SOCS5* belongs to the suppressor of cytokine signaling family, recognized as a STAT inhibitor protein family (13). This analysis identified genes that could be related to both canine and human melanomas.

Although canine oral melanoma has been studied for the past 70 years, the improvement of genomic and transcriptome analysis in the recent years allow for identification of markers that can be used with predictive or prognostic value.

#### Histological Aspects and Molecular Markers Associated With Aggressiveness in Melanomas, by Enio Ferreira

Regardless of the animal species affected, melanomas (malignant neoplasms of melanocytes) are aggressive neoplasms because of their high metastatic potential. In dogs, melanomas represent ~7% of all malignant tumors (14). The most common primary sites are the oral cavity, skin, mucocutaneous junctions, paws (nail junction and cushions), and, more rarely, the eyeball and meninges (15). Histopathological evaluation of melanomas can assist in both: the differential diagnosis of benign lesions and the prognostic determination. The mitotic index, nuclear atypia, and the degree of pigmentation stand out as relevant prognostic markers for melanomas. In addition to these, analysis of vascular invasion, junctional activity, and ulceration are also cited as determining prognostic factors in both cutaneous and oral melanomas by some authors (16, 17). There is no evidence that the histological type may be a relevant prognostic factor. The use of molecular markers (MELAN-A, PNL-2, HMB45, TRP-1, and TRP-2) is suggested for assisting the diagnosis of amelanotic melanomas, as these proteins are expressed in most of such tumors. Moreover, the analysis of Ki-67 expression is decisive in the prognostic definition of melanomas, but its analysis is dependent on the primary site of involvement (18). The expression of COX-2, KIT, metalloproteinases, EGFR, SOX, and proteins related to the epithelium-mesenchymal transition has been well-explored in canine melanomas (19–21). These proteins have a direct relationship with a more aggressive histological behavior, determined by the histopathological characteristics mentioned above. The analysis of gene mutations in melanomas in veterinary medicine is still not well-understood. Some changes have already been identified in the NRAS gene, PTEN KIT, and ERK1/2; however, their relevance is still uncertain (22). Clinical studies are necessary to determine the prognostic and predictive value of protein expressions and mutations in canine melanomas.

### Conventional Therapies for Canine Melanoma

#### Oncological Surgery and Its Limitations on the Treatment of Melanoma in Dogs, by Jorge Castro

Surgery is the gold standard of treatment and the most common one for the local management of all melanomas, including oral (23), cutaneous, and digital melanomas (24). Surgical resection with wide margins has always been one of the recommended approaches for oral melanoma in dogs, associated with regional lymphadenectomy. The more aggressive resections involving the maxillary bone (maxillectomies) can be associated with temporary ligation of the carotid artery. This is an adjuvant surgical technique that the literature contemplates as a surgical option with a lower loss of blood. Although surgical treatment is the main choice, Liptak and Withrow (25) and Boston et al. (23) recommend that because of its high metastatic potential, systemic therapy should also be considered as a therapeutic option for melanomas.

Surgical resections with wide margins, including 2–3 cm of bone margins and 1 cm of soft tissue margins, were performed in 70 cases of canine oral malignant melanomas (26). Histopathological analysis showed that 72.9% of the tumors were completely excised, with 10% of these patients showing local tumor recurrence. In this study, dogs that had surgery as the only treatment option had a progression-free interval >567 days and a mean survival time (MST) of 874 days. In the same year, Boston et al. (23) obtained 79.3% (73/92) success rate in complete excisions between surgeries performed with wide margin, based on histological evaluation, and the recurrence rate was 8.3% (6/73) in this group (23). The average survival time for these dogs was 354 days.

The resection of regional lymph node neoplasms in the face and maxilla region has been studied and performed since the last century (27) and a more recent study has mapped these lymphatic centers (28). The analysis of sentinel lymph nodes provides essential information about the clinical stage of the disease and helps to determine the most appropriate treatment plan (29, 30). In a survey conducted by Williams and Packer with 100 dogs with oral malignant melanoma, 53% had cytological or histopathological evidence of metastasis in mandibular lymph nodes, even in normal-sized lymph nodes (31). Therefore, lymphatic toilet (removal of regional lymph nodes) is recommended, especially in dogs with oral melanomas. In digital melanomas, lymphatic drainage in the limbs must also be evaluated and the removal of the regional lymph node in surgical planning is advocated (30, 31).

#### Radiation Therapy in Dogs With Oral Melanomas, by Simone C. S. Cunha; Natália Oyafuso Da Cruz

Radiation therapy (RT) is a localized cancer treatment modality, which acts on proliferating cells that fall inside the radiation field. In cases of canine oral melanoma, RT can be used as an adjuvant post-operative or a palliative therapy. Radiation field should, whenever possible, include regional lymph nodes, regardless of whether they have macroscopic alterations suggestive of regional metastasis. The main radiation protocols for melanoma are based on hypofractionation (3–6 RT fractions once or twice weekly), due the alpha/beta ratio range of 0.5–2.5 Gy. These ratios are similar to those of late-responding normal tissues, which are preferentially damaged by radiation delivered in large doses per fraction (32, 33). Side effects are usually mild, self- limiting, and confined to radiation field, and may include alopecia, skin hypo- or hyperpigmentation, dry radiodermatitis, and oral mucositis. The overall response rate of oral melanoma in previous literature is reported to be 75–85%, including complete and partial tumor responses, and the mean survival time is 230–363 days (34–36). In the first author's (SCSC) experience, radiation therapy is routinely used as an adjunctive post-operative therapy (in cases of incomplete surgical resection) or, more commonly, as a palliative treatment (in unresectable tumors or surgery declined by the dog owners). The high rate of distant metastasis (44–58%) remains the significant limiting factor for curative intention treatment of canine oral melanoma (34, 37, 38). The author Cunha et al. (39) evaluated the survival of 24 dogs with oral melanoma treated with orthovoltage radiotherapy. The mean age and standard deviation was 13 ± 2.6 years and the overall response rate was 93%, including 64% partial (one stage I, one stage II, seven stage III, and one stage IV) and 29% complete tumor responses (one stage III and three stage IV). Mean survival time was 390 days in stage I, 286 days in stage II, 159 days in stage III, and 90 days in stage IV. There was no influence of sex and age in RT responses. In conclusion, Radiation Therapy can be an interesting alternative treatment for canine oral melanoma, leading to reduction of tumor volume and pain relief, with mild side effects.

### Innovative Therapies for Canine Melanoma

#### Viral Vectors in Melanoma, by Bryan Strauss

Currently, immunotherapy has emerged as the most successful usage of viral vectors applied for gene therapy of melanoma, including IMLYGIC and chimeric antigen receptor T cells (CAR-T cells) (40, 41). In either case, the goal of the gene transfer approach clearly involves oncolysis (therapeutic destruction of tumor cells) associated with or due to the activity of immune cells. The work of the Viral Vector Laboratory (Instituto do Câncer do Estado de São Paulo, Faculdade de Medicina, Universidade de São Paulo) shares this objective and has developed a non-replicating adenoviral vector, AdRGD-PG, with improved mechanisms of transduction because of the inclusion of the RGD peptide in the virus fiber protein and high-level transcription of the therapeutic genes under the command of a p53-responsive promoter, termed PG (42, 43). Since melanoma cells frequently retain wild-type p53 (44), this can be exploited to drive transgene expression and act as a tumor suppressor protein, when re-activated (45).

We used the AdRGD-PG platform for the transfer of p14ARF (alternate reading frame of the CDKN2a locus, p19Arf in mice, p14ARF in humans, and canines) to activate p53 and promote cell death. In addition, transfer of the interferon-β gene not only acts as an immune modulator, but also contributes to the activation of p53 (46). We used the B16F10 mouse model of melanoma to show that the combination of p19Arf and IFNβ gene transfer resulted in cooperative cell killing *in vitro* accompanied by the expression of immunogenic cell death (ICD) markers and *in vivo* where C57BL/6 mice revealed activation of a Th1 immune response (43–46). However, IFNβ is known to function in a species-specific manner (47, 48); therefore, it is imperative to test our approach in relevant models. Recent work using human cDNAs and established human melanoma cell lines produced a similar result, where cell killing was even more efficient and continued to be associated with the expression of ICD markers (49, 50). Strikingly, an *ex vivo* model using human SK-MEL-147 cells was used to show that cell death induced by our treatment activated human dendritic cells that caused priming of T cells to induce a cytolytic response on encountering naïve tumor cells (50). Thus, we have shown that our gene transfer approach promotes oncolysis and immune activation and can be considered as an immunotherapy. Currently, we are gearing up to examine the efficacy of our approach in veterinary cases of melanoma because of their relevance and similarity to human cases as well as the demand for efficient therapies for canines. For this, a set of AdRGD-PG vectors has been constructed encoding the canine cDNAs of p14ARF and IFNβ. In parallel, three cell lines were isolated from canine oral melanomas (unpublished results), characterized for their ability to grow in tissue culture as well as form subcutaneous tumors in BALB/c nude mice. These cell lines were shown to harbor wild-type p53 and show permissive transduction and reporter gene expression with our AdRGD-PG vectors (51). This model system is currently being tested and will serve as proof of concept before embarking on experimentation in spontaneous cases of canine oral melanoma. These critical steps have been taken to demonstrate the potential of our immunotherapy in a relevant model.

#### Immuno-Oncology in Canine Oral Melanoma, by Cristina De Oliveira Massoco

In the last 20 years, immunotherapy has gained prominence since this therapeutic modality has significantly increased the effectiveness of treatments and the survival rates of cancer patients. The discovery that cells of the immune system interact with tumor cells and eliminate them is not a totally new concept and was born in the middle of the nineteenth century with the experiments carried out by Coley on human patients (52). Several clinical observations about spontaneous tumor regression and evidence showing a positive correlation between the presence of lymphocyte in a tumor microenvironment and increased survival rate reveal an increasing interest in the cancer immunosurveillance theory.

Especially for malignant melanoma, control of the disease with classical therapies and clinical management remains challenging, and yet immunotherapy modalities seem to be promising. Such strategies include DNA-based canine melanoma vaccine, immunotherapy methods using dendritic cells, and (non) viral vectors to deliver gene products, among others that have been studied (53). Even though responding melanoma-bearing dogs may have a partial or complete regression, some patients who are treated with immunotherapy showed varying response rates within cohorts with the same malignancy (54). Studies have shown that the efficacy of the immune system, in an anti-tumor context, is limited by the characteristics of immune cells present in the tumor microenvironment. These are reprogrammed to an immunosuppressive profile determined mainly by tumor cells, thus generating fewer effective responses against tumors (55, 56).

Cancer cells actively employ various tactics to delay, alter, or even stop antitumor immunity. These mechanisms develop continuously during the progression of cancer and become more diverse and complex in late-stage cancers (57). Therefore, it is critical to identify tumor immunity in the microenvironment to determine the outcomes and responses to immunotherapy. Identifying specific defects in the antitumor immune response as infiltrated immune cell population analysis associated with other immune signatures in dogs could serve as a basis for future studies to choose patients who will benefit from immunotherapy for oral melanoma.

#### Immunotherapy in the Treatment of Patients With Melanoma: OncoTherad® Nano-Immunotherapy, by Wagner Fávaro

**Figure 5:**
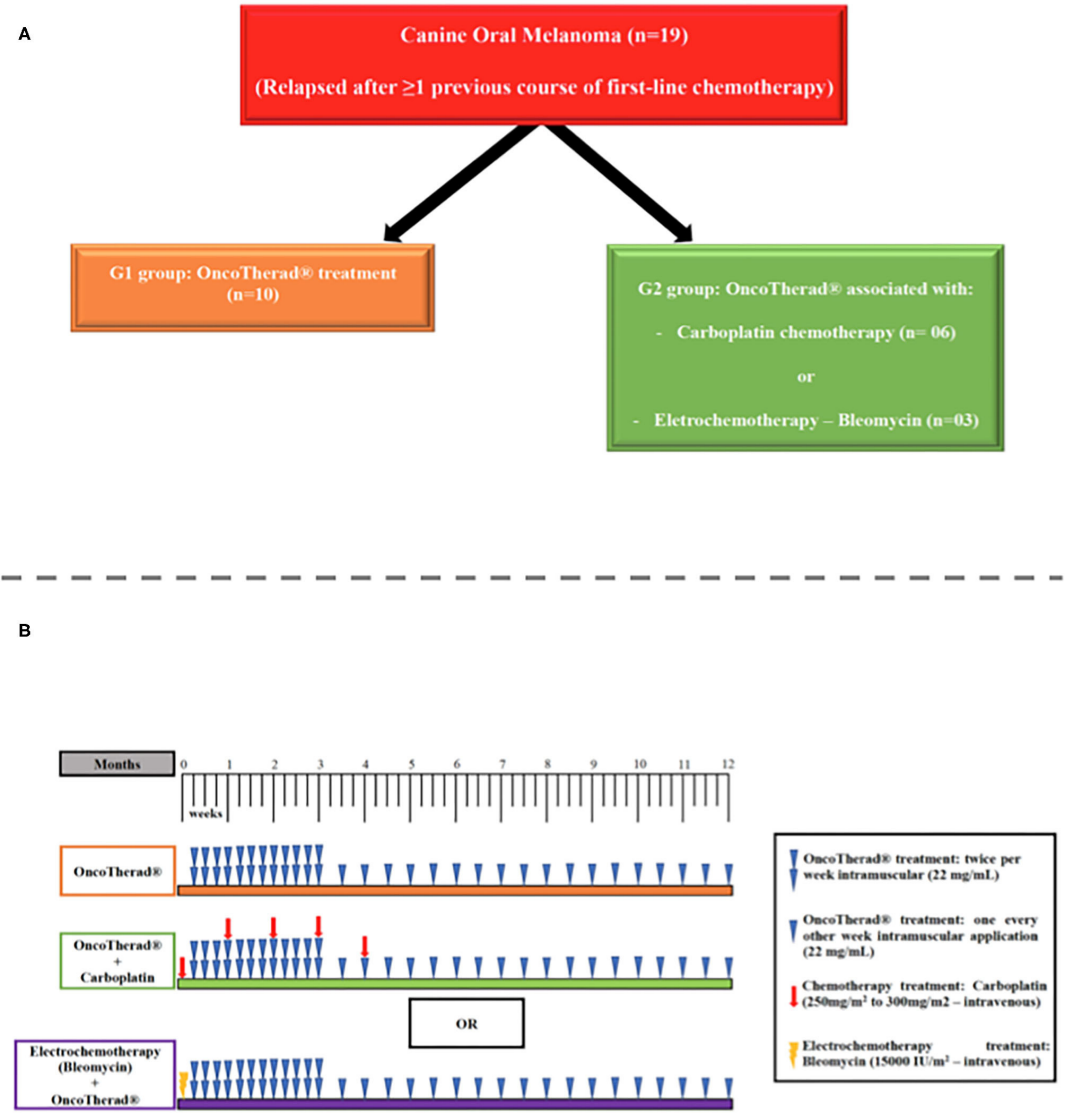
**(A)** Scheme of the group division (OncoTherad® treatment associated or not with chemotherapy or electrochemotherapy). **(B)** Timeline of the treatments (OncoTherad® treatment associated or not with chemotherapy or electrochemotherapy).

Canine oral melanoma (COM) is a highly aggressive and metastatic cancer. Many studies have demonstrated that post-treatment medium survival time ranges from 4.8 to 12 months (58). Conventional treatments have not shown suitable disease control partly because surgery rarely achieves complete resection, and chemotherapies require low tumor loads to be effective. Immunotherapy has become a promising cancer therapy, improving the prognosis of patients with many different types of cancer, and offering the possibility for long-term cancer remission. In this scenario, a new perspective is represented by OncoTherad® nano-immunotherapy. OncoTherad® is a nanostructured inorganic phosphate complex associated to glycosidic protein, developed by University of Campinas (UNICAMP), Brazil, which exhibits immunomodulatory and antitumor properties (59). OncoTherad® leads to a distinct stimulation of the innate immune system mediated by Toll-like receptors (TLRs) 2 and 4, resulting in an increased activation of the interferon (IFN) signaling pathway (60, 61). OncoTherad®-induced stimulation of the immune system via TLR2 and TLR4 occurs through the phosphorylation of hydroxylated amino acids, such as tyrosine, threonine, and serine by compounds that contain phosphate salts; this results in the activation of stimulator of interferon genes, with a consequent increase in the production of IFN-α and IFN-γ (62). The increase in the production of IFNs mediated by TLRs-2 and 4 promotes the activation of TCD8^+^ cells, dendritic cells, natural killer cells, and M1 macrophages, culminating in the induction of immunogenic cell death (62). Furthermore, OncoTherad® decreases the expression of receptor activator of nuclear factor-κB (RANK) and receptor activator of nuclear factor-κB ligand (RANK-L) system and, as a result, prevents or inhibits metastases progression (63). Thus, the aim of this study was to evaluate the efficacy of OncoTherad® nano-immunotherapy for first-line chemotherapy-relapse high-grade COM (with or without metastasis). We carried out a prospective, multicenter study in 19 (8 men, 11 women) consecutive patients with COM-relapse (≥1 previous course of first-line chemotherapy). OncoTherad® treatment (G1 group, *n* = 10) was initiated with its intramuscular (22 mg/mL) application twice per week for 3 months, followed by application, once every other week, until 1 year of treatment (Figure 5). OncoTherad® was associated (G2 group, *n* = 09) with chemotherapy (Carboplatin: 250—300 mg/m^2^—intravenous) or electrochemotherapy (bleomycin: 15,000 IU/m^2^—intravenous) (Figure 5). All canine patients received follow-up exams for 2 years. The Ethics Committee for Animal Experimentation (CEUA)/UNICAMP approved the animal procedures (protocol number 4861-1/2018). The median age of the 19 patients was 12.5 years (range 09–16) and follow-up time was 24 months. Based on iRECIST criteria, the overall complete response rate was 31.6% (G1 group−40.0%; G2 group−22.2%), overall partial response rate was 42.1% (G1 group−30.0%; G2 group−55.6%), and overall stable disease rate was 10.5% (G1 group−20.0%; G2 group−0.0%). Only 15.8% of total patients (G1 group−10.0%; G2 group−22.2%) presented progressive disease. The median overall progression-free survival was 640 days (G1 group−676.5 days, G2 group−599.4 days). In conclusion, OncoTherad® nano-immunotherapy seems an effective treatment option for chemotherapy-relapse COM patients and may provide benefits for preventing tumor progression.

#### Electrochemotherapy in the Treatment of Melanomas, by Marcelo Monte Mór Rangel

Electrochemotherapy is a new modality of local cancer treatment, which is increasingly gaining space, both in human and veterinary medicine. Its high index of objective response to different histological types, and its selectivity to cancerous tissue has encouraged its application in neoplasms that are more resistant to standard treatments. The main objective of surgery in the treatment of melanomas is the complete resection of the tumor and this usually occurs in wide surgeries. Large surgeries can be challenging in oral cavity melanomas and the need for adjuvant therapies aiming at better local control may be necessary. Electrochemotherapy is a local control technique with a high rate of objective response, selectivity to neoplastic tissue. Its use has been proposed in oral malignant melanomas that are difficult to resect, either as an adjunct to surgery or as a single treatment. The prior studies have shown promising results with singular use of the technique and its use as an adjunct with other therapies. These results corroborate the need for further studies to prove that the technique can be an alternative treatment for malignant melanomas in dogs (64–80).

#### Electrochemotherapy in the Treatment of Canine Oral Malignant mela noma, by Carlos Brunner

From July to December 2018, 58 melanomas in dogs were treated with electrochemotherapy with bleomycin and electroporation with BK100 electoporator, at the Universidade Paulista, UNIP, in São Paulo, SP, Brazil. The samples were composed as follows: a total of 54% of them were males, with an average age of 11.4 years. There was a higher prevalence of the following breeds: Yorkshire (10.7%), Golden Retriever (8.9%), Poodle (7.4%), Teckel (5.3%), and SRD (5.3%), similar to data found in literature. The incidence of oral melanomas was higher (75%), which was in contrast to the results of the literature review (62%). Bleomycin was used intra-lesionally at a dose of 1 U/cm^3^ or intravenously at a dose of 15 Y/m^2^, followed by electroporation with a BK100 pulse generator with a series of 8 pulses of 1 KV in 100 μs square waves each. This protocol has resulted in satisfactory survival rates after 2 years, including some complete remissions, in grade 1 and 2 melanomas. As described in classic literature, the anatomical location influences the biological behavior of melanomas; patients who underwent multimodal therapy, including surgery, conventional chemotherapy, and electrochemotherapy were the ones who obtained the best results.

#### Electrochemotherapy for the Treatment of Canine Melanoma: the Experience of Argentina, by Matías Tellado

For this round table, two cases of melanoma were presented. The results were evaluated according to the prognostic factors reported in previous work (81). The first patient was an 8-year-old, male cross-bred (mongrel) dog with stage I oral malignant melanoma located in the soft palate. No bone involvement was seen as determined by computed tomography scan. It was treated with BIOTEX EPV-200 (BIOTEX SRL, Buenos Aires, Argentina) electroporator, using 20 G disposable needles electrode. The device delivered 8,100 μs long square pulses of 1,000 V/cm at 5 kHz according to ESOPE 2018 standards (82). A month after the electrochemotherapy session, a complete response was obtained, and the patient remained disease free 11 months later and until the writing of this work. The second case was a 9 year-old female boxer with a stage IV oral malignant melanoma located in the right angle of the mandible, that extended to the soft palate and mucosa. No bone involvement was seen as assessed by CT scan. The patient had a small single pulmonary metastasis determined by chest radiograph. The treatment consisted of surgical excision of the oral mass, followed by ECT in the tumoral bed and margins. A month after the ECT treatment, the patient presented a complete local response. The local response and the lung metastasis remained unchanged until 5 months later. The results obtained were in accordance with previous published results regarding predictive factors of response to ECT in oral melanoma (80, 81). Early stages and absence of bone involvement displayed good response rates. In the second case, even if it was in an advanced stage, the combination with surgery to reduce the tumoral burden and the absence of bone involvement allowed us to expect a good outcome of the treatment.

#### Clinical Reports of Melanoma Undergoing Electrochemotherapy or Calcium Electroporation, by Denner Santos Dos Anjos

Multimodal therapies have been used in melanomas, such as surgery, radiotherapy, chemotherapy, immunotherapy, electrochemotherapy, and electro gene therapy. ECT is an effective local treatment that combines the administration of chemotherapeutic drugs, such as bleomycin or cisplatin, followed by the delivery of permeabilizing electrical pulses. In veterinary medicine, electrochemotherapy has been widely used because of its high efficacy in all solid tumors, such as cutaneous and subcutaneous tumors, skin metastasis, melanoma, sarcomas, and visceral tumors, such as thymoma and bladder cancer (83–89). In addition to bleomycin and cisplatin being the chemotherapeutic drugs mainly used in electrical pulses, carboplatin may also be a good candidate for electric pulses based on our clinical experience (Dr. Denner dos Anjos). Moreover, calcium electroporation (CaEP) is a novel therapeutic perspective for patients with cutaneous metastasis of MM and remission of metastatic melanoma foci. Its approach leads to supraphysiological calcium influx into neoplastic cells leading to acute ATP depletion killing cancer cells by necrosis and release of dangerous cellular signals boosting immunologic system locally. We have observed some patients diagnosed with MM stage III (tumor size more than 4 cm, negative lymph node) who underwent ECT with carboplatin plus CaEP had a disease free-interval of >720 days after one session or ECT with carboplatin and locally administered cisplatin (1 m/cm^3^) had a disease free-interval of >365 days. Furthermore, neoadjuvant ECT can also be used for partial remission in order to perform a better surgical approach for patients with advanced disease. To conclude, ECT may be an option for local control in regions where anatomic limitation is a challenge for wide excision.

## Discussion and Conclusion

The goal of scientific meetings and workshops is to call attention to new achievements in the field to ensure advancement of science and applications. In conclusion, this colloquium provided important information about canine melanomas. The Brazilian Association of Veterinary Oncology, ABROVET, hopes that this report can help veterinary oncologists from all over the world to better diagnose and treat these aggressive neoplasms in dogs.

## Author's Note

The Brazilian Association of Veterinary Oncology, ABROVET, is a non-profit organization that was created in 2004 by veterinary oncologists from Brazil (https://abrovet.org.br/). ABROVET frequently organizes scientific and cultural events and seeks to develop the field of Veterinary Oncology in Brazil. In 2016, ABROVET organized the 3rd. World Veterinary Cancer Congress in the city of Foz do Iguaçu, Brazil. The Colloquium on Canine Melanoma was an online event held in December 2020, and had around 200 participants.

## Author Contributions

CF-A, ÊF, CO, BS, WF, ND, NO, SC, JC, MR, CB, MT, DA, and SF: writing and editing the manuscript. AB and LB: mentoring and editing the manuscript. MLZD: mentoring, writing, and editing the manuscript. All authors contributed to the article and approved the submitted version.

## Conflict of Interest

The authors declare that the research was conducted in the absence of any commercial or financial relationships that could be construed as a potential conflict of interest.

## Publisher's Note

All claims expressed in this article are solely those of the authors and do not necessarily represent those of their affiliated organizations, or those of the publisher, the editors and the reviewers. Any product that may be evaluated in this article, or claim that may be made by its manufacturer, is not guaranteed or endorsed by the publisher.
